# Macrophage accumulation on the injured pulmonary surface promotes intrathoracic adhesions

**DOI:** 10.1038/s41392-026-02762-w

**Published:** 2026-06-02

**Authors:** Yu Mori, Tomohisa Sakaue, Mikio Okazaki, Mie Kurata, Hironori Izutani

**Affiliations:** 1 Department of Cardiovascular and Thoracic Surgery, Ehime University Graduate School of Medicine, Toon, Ehime Japan; 2 Department of General Thoracic Surgery and Breast and Endocrinological Surgery, Dentistry and Pharmaceutical Sciences, Okayama University Graduate School of Medicine, Okayama, Japan; 3 Department of Analytical Pathology, Ehime University Graduate School of Medicine, Toon, Ehime Japan

**Keywords:** Experimental models of disease, Translational research

**Dear Editor**,

Intrathoracic adhesions (IAs) often complicate thoracic surgery, increasing the risk of intraoperative bleeding, prolonged operative time, and postoperative air leakage.^1^ In addition, pleurodesis—an intentional induction of IAs—plays an important therapeutic role in the treatment of spontaneous pneumothorax; notably, 41.4% of patients with recurrent pneumothorax undergo either chemical or mechanical pleurodesis.^2^ Therefore, elucidating the molecular mechanisms underlying IA formation is crucial for optimizing both surgical management and therapeutic interventions. Although a pericardial adhesion model in mice has been reported,^3^ a simple murine model to understand the molecular mechanisms underlying adhesion between lung tissue and the chest wall for the development of therapeutic agents has yet to be developed. The present study aimed to establish a simple IA murine model to assess pathological changes and to identify potential targets for early preventive intervention against adhesions between the lungs and surrounding tissues.

A macroscopic image of the injection of adhesion inducers is presented in Fig. 1a, left. The macroscopic IA grading was evaluated at three locations within the intrathoracic cavity based on the scoring methods detailed in the Supplementary materials. The administration site was identified as the area between the left lung and chest wall (marked with an asterisk), visible through the diaphragm (Fig. 1a, left). Nine days after administration, talc-treated mice exhibited severe adhesions between the left lung and the adjacent chest wall tissues (Fig. 1a), although no signs of significant pain, distress, or mortality were observed. Despite the presence of a thin and incomplete mediastinum in mice, which may permit communication between the left and right pleural cavities, administration of talc into the left pleural cavity at a dose of 2 g/kg consistently induced reproducible intrathoracic adhesions between the left lung and chest wall. Hematoxylin and eosin, Masson’s trichrome staining, and immunohistochemical staining for COL1A1 showed that newly formed fibrotic layers, characterized by spindle-shaped cell infiltration and distinctly stained blue, were detected between the lung tissue and chest wall on day 9 (Fig. 1a, left). To determine the turning point at which significant lung adhesion was established, we evaluated and quantitatively scored the adhesion grade over a 21-day period post-talc administration. By day 9 post-administration, pronounced IA formation was observed with significantly high adhesion scores, which subsequently plateaued (Fig. 1a, right). Conversely, only mild adhesions were seen on days 3 and 6 (Fig. 1a, right). These results indicate that extracellular matrix (ECM) production on the injured pulmonary surface was primarily initiated from day 3 post-administration, and fibrotic tissues connecting the pulmonary tissue and chest wall were completely formed by day 9 post-administration, resulting in strong IAs characterized by a significantly high adhesion score. This mouse model provides a valuable tool for elucidating the molecular mechanisms underlying postoperative adhesions between lung tissue and adjacent tissues following thoracic surgery.

**Fig. 1:**
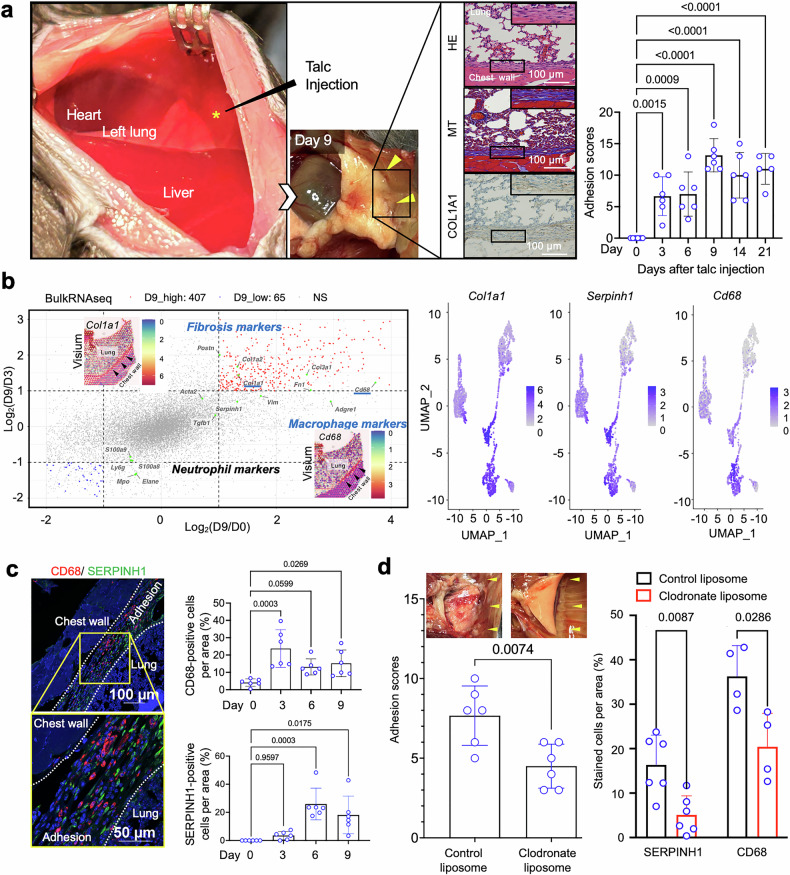
**a** Establishment of an intrathoracic adhesion (IA) model in mice. (Left) Enlarged left thorax view following laparotomy; the asterisk (*) represents the injection point of adhesion inducers. Representative macroscopic findings and IA formation at 9 days post-injection. Hematoxylin and eosin (H&E), Masson’s trichrome, and immunohistochemical staining for COL1A1 in tissues 9 days after adhesion induction in the region delineated by the black rectangular outline in the gross image. Scale bar = 100 μm. (*Right*) Quantification of adhesion scores at 3, 6, 9, 14, and 21 days post-injection (*n* = 5–6 for each group). Statistical significance was determined using Dunnett’s multiple comparisons test. Data are represented as mean ± SD. **b** Integrated spatial transcriptomics and bulk RNA-seq analysis for unveiling the molecular mechanism underlying IA formation. (*Left*) Multi-dimensional scatter plot of Log_2_(D9/D3) versus Log_2_(D9/D0) for bulk RNA sequencing at day 0 (D0), day 3 (D3), and day 9 (D9). Data analysis was performed using the RNAseqChef web tool (https://imeg-ku.shinyapps.io/RNAseqChef/). NS, not significant. The plot highlights neutrophil markers (*Ly6g, Mpo, Elane, S100a8*, and *S100a9*), macrophage markers *(Cd68 and Adgre1*), and fibrosis markers (*Col1a1, Col3a1, Acta2, Fn1*, and *Postn*). Spatial-transcriptomics (Visium) for *Col1a1* and *Cd68* corresponding to H&E-stained sections harvested from mice 6 days after talc injection are embedded in the scatter plot. The regions indicated by black arrows show the adhesion sites between the chest wall and lung tissue. (*Right*) UMAP plots showing the expression of *Col1a1, Serpinh1, and Cd68* generated from spatial transcriptomics data. **c** Macrophage accumulation on the surface of lung tissue prior to IA formation. (Left) Immunofluorescence staining with anti-CD68 (*red*) and anti-SERPINH1 (*green*) antibodies for detecting macrophages and collagen-producing cells, respectively, between the lung parenchyma and chest wall in IA models. (Right) Quantitative comparison of CD68- and SERPINH1-positive cells per area (%) in a randomly selected area of IA, viewed at 400× magnification (*n* = 6 for each group). Quantitative data were obtained from immunohistochemical staining data. Statistical significance was determined using a Kruskal–Wallis test. Data are represented as mean ± SD. **d** Macrophage depletion effectively inhibits IA formation. (Left) Quantification of adhesion scores of mice in the control and macrophage-depleted groups on day 6 (*n* = 6 for each group). Statistical significance was assessed using Student’s *t* test (*p* = 0.0074). Data are represented as mean ± SD. Macroscopic intrathoracic images of mice in the control and macrophage-depleted groups on day 6. (Right) Quantification of immunohistochemical staining with anti-SERPINH1 antibodies on mouse tissue from the control and macrophage-depleted groups, focusing on the pleural surface on day 6 (*n* = 6 for each group). Statistical significance was determined using a Mann–Whitney test. Data are represented as mean ± SD. Quantification of CD68-positive macrophages per area (%) in control and macrophage-depleted mice on day 3 (*n* = 4 for each group). Statistical significance was determined using a Mann–Whitney test. Data are represented as mean ± SD

Further investigations were conducted to understand the mechanisms underlying IA formation by comparing gene expression before and after IA formation using bulk RNA-sequencing analysis and spatial transcriptomics. Bulk RNA-sequencing showed that in total, 407 genes were significantly upregulated and 65 genes were significantly downregulated on day 9 compared with days 0 and 3. Among them, the expression of fibrosis-related markers, including *Fn1*, *Col1a1*, *Col3a1*, and *Postn*, as well as the macrophage marker *Cd68*, was significantly increased on day 9, based on the criteria of p < 0.05 and |log₂ fold change | >1 (Fig. 1b). At the acute stage (day 3), higher expression levels of neutrophil markers, including *S100a9 and S100a8*, were observed. At a later stage (day 9), genes related to the ECM inducer and its production, such as *Tgfb1* and *Serpinh1* were upregulated (Fig. 1b, upper left). Based on the results of spatial transcriptomics, on day 6 after adhesion induction, *Col1a1* and *Cd68* mRNAs showed high expression levels at the adhesion sites between the lung tissue and the thoracic wall (Fig. 1b, left, arrowheads). Next, we performed a more detailed spatial transcriptomic analysis to visualize collagen-producing cells that mediate adhesion between the lung tissue and the chest wall. Uniform manifold approximation and projection (UMAP) analysis revealed that the expression patterns of *Col1a1*, a fibroblast marker, closely resembled those of *Serpinh1*, indicating that Serpinh1 serves as an intracellular marker of collagen-producing cells, whereas the expression pattern of *Cd68* was distinct from those of these two genes (Fig. 1b, right). These data suggest that immune cells, particularly macrophages, may contribute to the recruitment of SERPINH1-positive cells and promote collagen production, thereby facilitating adhesion formation between the chest wall and lung tissue. To test this hypothesis, we examined the tissue distribution of SERPINH1-positive collagen-producing cells and CD68-positive macrophages. Both cell types were localized within the adhesion tissue. CD68-positive macrophages accumulated extensively between the chest wall and lung tissue during the acute phase of adhesion induction and subsequently declined (Fig. 1c). In contrast, SERPINH1-positive cells increased from day 6 onward (Fig. 1c) and were localized in close contact at the interface between the chest wall and lung tissue (Fig. 1c). Next, to determine the functional role of CD68-positive macrophages in IA formation, we depleted these cells by injecting clodronate liposomes. Clodronate liposomes significantly reduced IA formation in mice, whereas prominent adhesions between the lung tissue and chest wall were consistently observed in control liposome-treated mice (Fig. 1d, left). Clodronate liposome treatment significantly reduced adhesion scores and markedly suppressed the accumulation of SERPINH1-positive collagen-producing cells following CD68-positive cell depletion (Fig. 1d, right). These findings indicate that preventing macrophage accumulation on the lung surface represents a critical intervention point for inhibiting adhesion formation (Fig. 1d). Our results agree with evidence that foreign body–induced lung fibrosis is macrophage-dependent^4^ and suggest that GATA6⁺ macrophages participate in early injury responses, which may influence subsequent adhesion formation.^5^ The simple and reproducible mouse model presented herein—integrated with molecular profiling—provides a powerful platform for elucidating IA pathology and accelerating the development of preventive therapeutics. Nevertheless, further studies are warranted to more thoroughly examine the differences between this model and human pathology, as well as to clarify the mechanisms underlying macrophage accumulation at adhesion sites. Taken together, these findings indicate that following injury, macrophages accumulate on the surfaces of the lung and chest wall and play a central role in adhesion formation. Importantly, our results indicate that collagen production by fibroblasts is a secondary event in adhesion, whereas macrophages serve as the primary and initiating drivers of the fibrotic response. Macrophage accumulation precedes and instructs the activation of SERPINH1-positive fibroblasts, thereby triggering ECM deposition and subsequent tissue adhesion. Elucidating the molecular mechanisms that govern macrophage accumulation may enable the development of macrophage-guiding strategies, potentially allowing controlled adhesion formation without reliance on talc-based formulations. Accordingly, macrophage depletion reduces the number of SERPINH1-positive cells and fibrosis, thereby suppressing tissue adhesion.

## Supplementary information

SUPPLEMENTAL_INFORMATIONS

## Data Availability

The RNA-Seq data were deposited in the NCBI Sequence Read Archive (SRA) under the accession number GSE284182. Spatial-transcriptomics data were deposited in GSE308260.
