# Supplementary material for: Macrophage accumulation on the injured pulmonary surface promotes intrathoracic adhesions
Source: Signal Transduct Target Ther. 2026 Jun 2;11:209. doi: 10.1038/s41392-026-02762-w (PMC13230984; doi:10.1038/s41392-026-02762-w)
Supplement: Supplementary file 1 — SUPPLEMENTAL_INFORMATIONS [file 41392_2026_2762_MOESM1_ESM.docx]

Supplementary Materials for

Macrophage accumulations on the injured pulmonary surface promote intrathoracic adhesions

Yu Mori^1^, Tomohisa Sakaue^1*^, Mikio Okazaki^2^, Mie Kurata^3^, and Hironori Izutani^1*^

^*^Correspondence to: [sakaue@m.ehime-u.ac.jp](mailto:sakaue@m.ehime-u.ac.jp); [izutani@m.ehime-u.ac.jp](mailto:izutani@m.ehime-u.ac.jp)

**This file includes:**

Materials and Methods

**Material and Methods**

**Murine IA model**

All animal experiments were approved by the Ehime University Animal Care Committee (Project number: 05-RO-13-1). Eight-week-old C57/Black6 (B6) male mice weighing approximately 20 g were obtained from CLEA Japan (Tokyo, Japan). All mice were housed at 24 ± 1 °C with an automatically controlled light cycle from 07:00 to 19:00 h. The mice were sedated with a mixed injection of ketamine (100 mg/kg) and xylazine (10 mg/kg) in the intra-abdominal cavity. After making an incision in the skin and peritoneum, either 200 μL of a saline-based talc solution (2.0 g/kg; Unitalc; Nobelpharma, Tokyo, Japan) were administered into the left thoracic cavity through the diaphragm with a 24-gauge needle. The mice were sacrificed to evaluate IAs at 30 min (day 0) and 3, 6, 9, 14, and 21 days post-administration, and the whole thorax tissue containing the heart, left lung, and chest wall was excised. For histological analysis, the specimens were fixed with 4% paraformaldehyde for 24 h and embedded in paraffin. For gene expression analyses, samples were immediately homogenized with phenol solution after the mice were euthanized via cervical dislocation under anesthesia.

**IA scoring**

The macroscopic IA score was determined based on the sum of adhesion strength and area at three points in the thoracic cavity (the lung adjacent to the pericardium, diaphragm, and chest wall), as follows. This grading system evaluates intrathoracic adhesions by extent and strength, each scored from 0 to 3. Extent is graded as none (0), involving less than one-third of the area (1), less than two-thirds (2), or two-thirds or more (3). Strength is graded as none (0), easily separated with cotton (1), separated with cotton and partly with scissors (2), or only separable with scissors (3). The method for scoring adhesion tissues was based on previously published studies.^1^

**Histopathological findings and immunostaining**

The excised specimens were fixed with 4% paraformaldehyde (Wako) overnight and then cut in the axial direction, including the heart, left lung, adhesion tissue, and chest wall. Specimens were embedded in paraffin and sliced into 5-μm sections. H&E and Masson's trichrome staining were performed to visualize the tissue structure and fibrosis, respectively, following previously reported protocols.^1,2^ Bright-field images were acquired using a microscope (BX51, Olympus, Tokyo, Japan). To visualize the localization of COL1A1, tissues were deparaffinized with xylene and rehydrated with 70% ethanol and tap water for 20 min. For antigen activation, tissues were heated for 15 min at 120 °C with 10 mM sodium citrate buffer (pH 6.0) using an autoclave. Following the inactivation of endogenous peroxidase with a 3% hydrogen peroxide solution (Wako), the tissues were treated with the blocking reagent Dako REAL^TM^ Antibody Diluent (Dako, Carpinteria, CA, USA) for 30 min. The tissues were then incubated with anti-Col1a1 antibody (1:1000; #72026, Cell Signaling Technology, Massachusetts, USA) overnight at 4 °C. Subsequently, tissues were washed with phosphate-buffered saline three times and incubated with an anti-rabbit secondary antibody (#414341, Nichirei Biosciences Inc., Tokyo, Japan) for antigen–antibody reaction for 45 min at 25 °C. Antigens were stained with a DAB substrate solution (#S2022, Agilent, California, USA). After staining the nuclei with hematoxylin, the tissue sections were covered with a glass slip, and images were acquired as previously described. For visualization of two proteins, immunofluorescent co-staining was performed. Following antigen retrieval, samples were blocked with antibody diluent for 30 min. Tissues were incubated overnight at 4 °C with a mixture of primary antibodies, including anti-CD68 (1:400, #97778, Cell Signaling Technology), together with anti-SERPINH1 (1:400, ab109117, Abcam, Cambridge, UK). After washing, samples were incubated with Alexa Fluor 488–conjugated anti-mouse (1:1000, Thermo Fisher Scientific, Cat. No. A-11001) and Cy3-conjugated anti-rabbit (1:1000, Thermo Fisher Scientific, Cat. No. A-10502) secondary antibodies. Nuclei were counterstained with Hoechst 33258, and sections were mounted using ProLong Gold Antifade Mountant (Thermo Fisher Scientific, Cat. No. P36930). For quantification of CD68 and SERPINH1 proteins in murine tissues, DAB staining was performed as previously described. Briefly, tissue sections were deparaffinized, subjected to antigen retrieval, and blocked, followed by overnight incubation at 4 °C with either an anti-CD68 antibody (1:500, #97778S, Cell Signaling Technology, MA, USA) or an anti-SERPINH1 antibody (1:1000, ab109117, Abcam, Cambridge, UK). Sections were then incubated with an anti-rabbit secondary antibody (#414341, Nichirei Biosciences Inc., Tokyo, Japan) for 45 min at 25 °C. Antigen detection was performed using a DAB substrate solution (#S2022, Agilent, CA, USA). After nuclear counterstaining with hematoxylin, sections were mounted with coverslips, and images were acquired as previously described. Quantification was performed by calculating the occupancy ratio of CD68- or SERPINH1-positive cells, defined as the number of marker-positive cells divided by the total number of cells within 100 µm of the lung surface.

**RNA-sequencing analysis**

IA tissues, including the left lung parenchyma and chest wall, were harvested as a 6-mm square tissue specimen centered on the lung–chest wall interface and homogenized using QIAZol lysis reagent. Total RNA was isolated using a QIAGEN miRNeasy Micro Kit (QIAGEN, Hilden, Germany) following the manufacturer's protocol. The RNA concentration and integrity were assessed using a NanoDrop 2000 spectrophotometer (Thermo Fisher Scientific, Waltham, MA, USA) and an Agilent Bioanalyzer RNA 6000 Nano Assay Kit (Agilent Technologies), respectively. Total RNA was extracted, and strand-specific RNA-seq libraries were prepared by Rhelixa Co., Ltd. (Tokyo, Japan) using the NEBNext® Poly(A) mRNA Magnetic Isolation Module and the NEBNext® Ultra™ II Directional RNA Library Prep Kit, based on a dUTP-based method. Libraries were sequenced on an Illumina NovaSeq 6000 platform to generate 150-bp paired-end reads (PE150), yielding approximately 4 Gb of data per sample, corresponding to an average of 26.7 million reads (13.3 million read pairs) per sample. FASTQ files were generated and subsequently trimmed and mapped using CLC Genomics Workbench software (QIAGEN). Differential gene expression and enrichment analyses were performed using RNAseq Chef version 1.1.2 (<https://imeg-ku.shinyapps.io/RNAseqChef/>).^3^ Spatial transcriptomic profiling was performed using the Visium CytAssist platform (10x Genomics). Library preparation and sequencing were outsourced to Dr. Daisuke Motooka at the Research Institute for Microbial Diseases (RIMD), Osaka University, for support with the sequencing of CytAssist Visium libraries. At 6 days post-injection, mice were sacrificed 30 minutes after the final procedure, and thoracic tissues were immediately collected from the left lung parenchyma adjacent to the chest wall, fixed in 4% paraformaldehyde for 24 h, and sectioned at 5 μm for spatial transcriptomic analysis using the Visium Mouse Transcriptome Probe Set and Visium V4 Slide with probe-based v2 chemistry to extract total RNA and hybridize transcriptomic probes to the capture areas. Sequencing libraries were prepared according to the standard protocol described in the Visium CytAssist Spatial Gene Expression Reagent Kits User Guide. Paired-end sequencing (43 + 100 bp) was performed on the DNBSEQ-G400 platform, and raw sequencing data were processed using Space Ranger (version spaceranger-4.0.1, 10x Genomics) with alignment to the mm10-2020-A reference genome to generate gene–barcode count matrices. Processed outputs included filtered_feature_bc_matrix.h5 (HDF5 file containing filtered gene-by-spot counts), raw_feature_bc_matrix.h5 (HDF5 file containing unfiltered counts), and spatial.tar.gz (compressed directory containing spatial image and associated metadata). Subsequent analyses, including dimensionality reduction using Uniform Manifold Approximation and Projection (UMAP) and spatial expression heatmap visualization, were conducted in R (version 4.4.0) using the Seurat (version 5.1.0) and related packages. All analyses were performed on the raw count data without prior aggregation across spots, unless otherwise stated.

**Macrophage depletion**

To investigate the functional roles of macrophages during IA formation, mice were intraperitoneally administered 100 μL of clodronate liposomes or control liposomes (Catalog no. F70101C-AC, Formumax, California, USA) per 20 g of body weight. The following day, talc solution was administered into the left thoracic cavity for IA induction as mentioned above. On day 3 post-talc administration, the mice were sacrificed to evaluate the depletion of CD68-positive macrophages using immunohistochemical staining for CD68, as mentioned above. The mice were sacrificed on day 6 post-talc administration to quantify IA levels, followed by further histological analysis.

**Ethics statement**

All animal experiments were approved by the Ehime University Animal Care Committee (Project number: 05-RO-13-1) and were performed following the standards of the committee using approved animal protocols.

**Statistical analysis**

Data are presented as mean ± standard deviation. Statistical analyses were performed using GraphPad Prism version 10 (GraphPad Software, San Diego, CA, USA). One-way analysis of variance was used for normally distributed data, and the Kruskal–Wallis test for non-normally distributed data. For comparisons between two groups, Student's *t*-test was used for normally distributed data, and the Mann–Whitney test for non-normally distributed data. Differences between the two groups were considered statistically significant at *p* < 0.05.

**References**

1 Kojima, A. *et al.* A simple mouse model of pericardial adhesions. *J Cardiothorac Surg* **14**, 124 (2019). <https://doi.org/10.1186/s13019-019-0940-9>

2 Sakaue, T. *et al.* Proteomics-based analysis of lung injury-induced proteins in a mouse model of common bile duct ligation. *Surgery* **161**, 1525-1535 (2017). <https://doi.org/10.1016/j.surg.2016.12.017>

3 Etoh, K. & Nakao, M. A web-based integrative transcriptome analysis, RNAseqChef, uncovers the cell/tissue type-dependent action of sulforaphane. *J Biol Chem* **299**, 104810 (2023). <https://doi.org/10.1016/j.jbc.2023.104810>
